# Genome-Wide Association Study for Agro-Morphological Traits in Eggplant Core Collection

**DOI:** 10.3390/plants11192627

**Published:** 2022-10-06

**Authors:** Nayoung Ro, Mesfin Haile, Bichsaem Kim, Gyu-Taek Cho, Jungro Lee, Yoon-Jung Lee, Do Yoon Hyun

**Affiliations:** 1 National Agrobiodiversity Center, National Institute of Agricultural Sciences, Rural Development Administration, Jeonju 54874, Korea; mesfinhaile97@gmail.com (M.H.); bsam92@korea.kr (B.K.); gtcho@korea.kr (G.-T.C.); jrmail@korea.kr (J.L.); lyn3838@korea.kr (Y.-J.L.); 2 Department of Crops and Forestry, Korea National University of Agriculture and Fisheries, Jeonju 54874, Korea; dyhyun@korea.kr

**Keywords:** agro-morphological trait, eggplant, GBS, GWAS, SNP marker

## Abstract

Eggplant is one of the most economically and nutritionally important vegetables worldwide. The study of the association of phenotypic traits with genetic factors is vital for the rapid and efficient identification and selection of eggplant genetic resources for breeding purposes with desired traits. The eggplant resources (587) collected from different countries, including Korea, were used for establishing the core collection. A total of 288 accessions were selected from 587 *Solanum* accessions based on 52 single nucleotide polymorphisms (SNPs) markers together with 17 morphological traits. This core collection was further used to analyze the genetic associations of eggplant morphological variations. A large variation was found among the evaluated eggplant accessions for some agro-morphological traits. Stem prickles and leaf prickles showed a significant positive correlation (r = 0.83***), followed by days to flowering and days to maturity (r = 0.64***). A total of 114,981 SNPs were filtered and used for phylogenetic tree analysis, population structure analysis, and genome-wide association study (GWAS). Among the agro-morphological traits, significantly associated SNPs were found for six traits. A total of 377 significantly associated SNPs with six agro-morphological traits were identified. These six traits and the number of SNPs were: days to maturity (51), flower size (121), fruit width (20), harvest fruit color (42), leaf prickles (38), and stem prickles (105). The largest fraction of significant SNPs (11.94%) was obtained on chromosome Ch01, followed by Ch07 and Ch06 with 11.67% and 10.08%, respectively. This study will help to develop markers linked to the most important agro-morphological traits of eggplant genetic resources and support the selection of desirable traits for eggplant breeding programs.

## 1. Introduction

Eggplant (*Solanum melongena* L.), a member of the *Solanaceae* family, is a popular vegetable in Africa, Asia, and Southern Europe [1]. In India and China, eggplant is the third most important solanaceous crop after potatoes and tomatoes [2]. Eggplant is a reliable source of vitamins, minerals, and antioxidants in the human diet. Many of the breeding objectives of vegetable and fruit crops (mainly yield, resistance, or tolerance to biotic and abiotic stresses) are shared by the eggplant. However, there are some specific eggplant breeding traits that include aiming to develop prickleless (stem, leaf, and calyx) eggplant varieties and reduce fruit bitterness [3].

The availability of diverse genetic materials is critical for the development of new crop varieties [4]. Crops with a narrow genetic basis are vulnerable to new diseases and other constraints that reduce production, which can result in significant declines in areas of adaptation [5]. It is becoming increasingly important to develop new eggplant varieties with higher yields and improved agronomic characteristics such as optimal plant architecture and fruit shape, low risk of deterioration during transport, and longer storability. Despite the economic importance of eggplant improvement, its genome has received less attention than that of closely related *Solanaceae* species: tomato, potato, and pepper [3]. However, eggplant breeders have recently begun using marker-assisted selection.

Linkage mapping has revealed the genetic basis of certain fruit and plant morphological traits in both intra-specific [3] and inter-specific [6,7,8] populations. In a pioneering attempt to apply a genome-wide association (GWA) approach, Ge et al. [9] were able to identify some phenotype-genotype associations for eight fruit-related traits. The identification of quantitative trait loci (QTL) associated with several agronomic traits has been developed in eggplant, as has the improvement of genetic linkage map construction. For anthocyanin pigmentation, fruit morphology (weight, length, diameter, metabolic content, and shape), and prickleless, for example, several QTLs have been identified using an intraspecific F2 population and a 238-loci linkage map [2,3,6,10,11]. However, when compared to other vegetable crops such as tomato and cucumber, the identification and characterization of QTLs and functional genes underlying important agronomic traits in eggplant has lagged significantly, owing in part to the lack of a genetic linkage map with high-density markers. So far, with the help of next-generation sequencing (NGS) technologies, four eggplant reference genomes have been published [12,13,14,15], which would greatly facilitate developing a large number of SNP markers for genetic map construction, resulting in improved efficiency of fine gene mapping.

GWAS is a powerful technique for deciphering the genetic basis of complex phenotypes by exploiting naturally occurring genetic variability [16]. GWAS enables the detection of relationships between molecular markers and desirable traits with better mapping resolution than standard bi-parental populations and has been used to identify markers associated with desired traits in a variety of crops [17,18,19]. GWAS involves an assessment of the population structure of the diversity panel to determine the genetic relatedness of individuals and rule out erroneous associations [16,20] and relies on the use of a sufficiently large number of markers. Recent advancements in next-generation sequencing technology and SNP genotyping have given breeders more tools for characterizing genetic variation at high resolution and selecting desired traits when developing new varieties.

Therefore, the purpose of this study was to characterize the phenotypic features of eggplant germplasm and identify SNP markers associated with the agro-morphological traits. In this study, the GWAS panel included a total of 288 eggplant germplasms from different species and significantly associated SNP markers for some agro-morphological features were identified.

## 2. Results

### 2.1. Phenotypic Variation and Correlations of Eggplant Core Collection

The eggplant resources (587) collected from 50 countries, including 80 resources in the Philippines, 44 resources in China, and 16 resources in Korea, were used for establishing the core collection. A total of 288 eggplant resources were selected from 587 *Solanum* accessions based on 52 SNP markers together with agro-morphological traits. The available phenotype data for 17 traits was included in the selection of a representative core collection because the core sets selected using only genotype data could not represent the diversity of the entire collection, presumably due to limitations in the number of SNP markers used. This core collection was further used for a genome-wide association study.

Phenotypic characterization of 17 qualitative and quantitative agro-morphological traits was performed (Table 1 and Table 2) for 288 germplasms. Of the eggplant collections evaluated, 260 accessions (90.28%) had an intermediate growth habit, 15 (5.21%) had an upright growth habit, and 13 (4.51%) had a prostrate growth habit. Most eggplants lacked anthocyanin pigmentation on the hypocotyl and fully developed stems. Also, the majority of the eggplant accessions had no prickles on the stem, leaf, or calyx. Regarding flower size, 33 (11.46%) accessions had small (2 cm) flowers, 250 (86.81%) had medium (2–3 cm) flowers, and 5 (1.74%) had large flowers. Flower colors were purple (61.11%), light purple (21.18%), white (16.32%), and white and purple (mixed) (1.39%). The predominant immature fruit colors of eggplant germplasm were green and purple with 38.19% and 36.11%, respectively. As for fruit color at maturity, purple (40.28%) and green (22.57%) were the two most common colors among eggplant germplasm. The majority of eggplant germplasms had light brown (tan) and yellow fruit at harvest (47.22% and 37.50%, respectively). Table 2 presents the minimum, maximum, averages, and standard deviations of quantitative agro-morphological data for 288 eggplant core collections. The plant height of eggplants ranged from 13.20 cm to 210 cm. The average plant height, fruit width, fruit length, days to flowering, and days to maturity were 87.76 cm. 5.77 cm, 16.80 cm, 110, and 156 days, respectively (Table 2).

**Figure 1:**
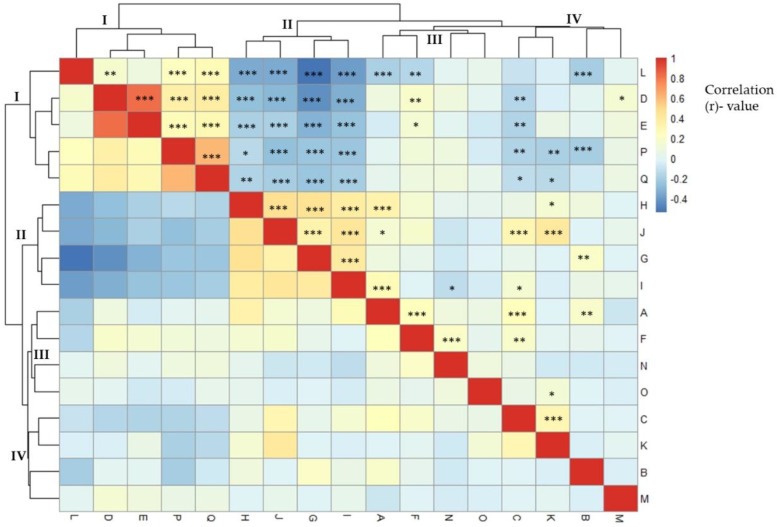
Pearson’s correlation of phenotypic traits of eggplant accessions in a clustered heatmap. (A: hypocotyl anthocyanin, B: growth habit, C: stem anthocyanin, D: stem prickles, E: leaf prickles, F: calyx prickles, G: flower size, H: flower color, I: fruit shape, J: immature fruit color, K: mature fruit color, L: fruit color at harvest, M: plant height, N: fruit length, O: fruit width, P: days to flowering, and Q: days to maturity). Significant correlation indicated with asterisks (* *p* < 0.05; ** *p* < 0.01; *** *p* < 0.001).

The correlation between agro-morphological characteristics is shown in Figure 1. Anthocyanin pigmentation of the hypocotyl and stem showed a positive correlation (r = 0.23 ***). Of 288 eggplant germplasm samples, 58 had pigmented hypocotyls and 230 did not. Similarly, a large number of accessions (202) lacked anthocyanin pigmentation on the stems, whereas the remaining 86 accessions had pigmented stems. There was a significant, strong positive correlation between stem prickles and leaf prickles (r = 0.83***). The majority of eggplant genetic resources did not have prickles on the stems (266 germplasms) or leaves (257 germplasms). A strong positive correlation (r = 0.61***) was found between days to flowering and days to maturity. As shown in Figure 1, the agro-morphological traits were grouped into four main clusters according to the correlation coefficient values. The first cluster (I) comprised five agro-morphological traits; fruit color at harvest, stem prickles, leaf prickles, days to flowering and days to maturity. There was a strong positive correlation among traits within the first cluster. The second cluster included flower color, immature fruit color, flower size, and fruit shape. The correlation within the second (II) cluster was positive and moderate. The third cluster (III) contained hypocotyl anthocyanin, calyx prickles, fruit length and fruit width, whereas the fourth cluster (IV) comprised stem anthocyanin, mature fruit color, growth habit and plant height. There was a moderate to high negative correlation between the traits of clusters I and II. The agro-morphological traits of clusters I and III had a weak positive to weak negative correlation, whereas clusters I and IV had a weak positive to moderate negative correlation. The correlation between clusters II and III traits was moderate positive to weak negative.

**Figure 2:**
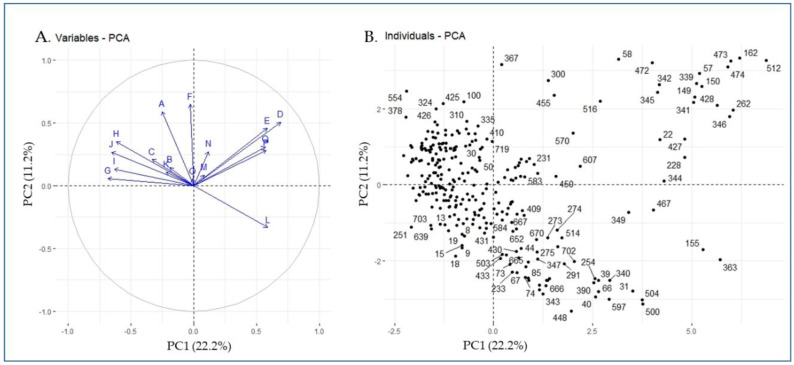
PCA plot based on the phenotypic data of 288 eggplant accessions (**A**: variables and **B**: individuals; each dot represents a single accession). Letters (A–Q) on the PCA plots indicate phenotype traits; A: hypocotyl anthocyanin, B: growth habit, C: stem anthocyanin, D: stem prickles, E: leaf prickles, F: calyx prickles, G: flower size, H: flower color, I: fruit shape, J: immature fruit color, K: mature fruit color, L: fruit color at harvest, M: plant height, N: fruit length, O: fruit width, P: days to flowering, and Q: days to maturity.

**Figure 3:**
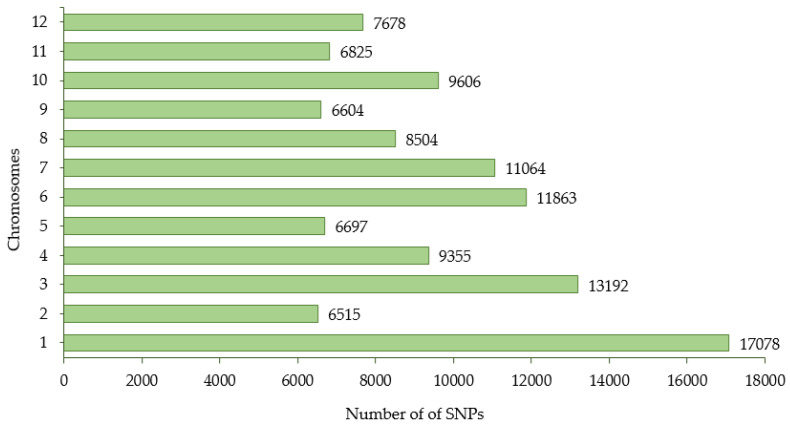
The distribution of SNPs generated from 288 eggplant genetic resources across 12 chromosomes.

Principal component analysis (PCA) plot was generated using the phenotypic data of 288 eggplant accessions (Figure 2). The first five PCs explained 57.6% of the total variance. PC1 accounted for 22.2% of total phenotypic variation. Stem prickles, immature fruit color, flower size, fruit shape, and flower color were the top five contributors of agro-morphological-related traits to PC1. Meanwhile, PC2, which was primarily associated with calyx prickles, hypocotyl anthocyanin, stem prickles, and flower color, explained 11.2% of the total variance. The positively and negatively correlated agro-morphological traits and the corresponding individual eggplant genetic resources are visualized in Figure 2A,B. The fruit color at harvest (L) was positively correlated and showed a wide distance from other variables (Figure 2A) and most of the germplasm (Figure 3) corresponded with fruit harvest color (code: 363, 155, 467, 349, 341, 504, etc.) had red-colored fruits at the ripening stage.

### 2.2. Genotyping-by-Sequencing and SNP Calling 

The GBS library was constructed from 288 eggplant accessions and sequenced using the Illumina Hiseq 2000 platform (Illumina, Madison, WI, USA) and generated approximately 2.2 billion reads with an average mapping depth of 25.41× for a single accession. Table 3 and Table 4 present a summary of these sequencing results. The summary of the reference genome, including chromosome length (bp), number of transcripts, transcript length (bp), and CDS length (bp) for each chromosome is presented in Supplementary Table S1. The genotyping of the eggplant core collection detected 1,859,683 SNPs covering 12 chromosomes. A total of 114,981 SNPs were obtained after filtering the frequency of minor alleles (>5%) and missing data (<30%) (Table 5). The number of SNPs retained on each chromosome is presented in Figure 3. 

### 2.3. Population Structure and Phylogenetic Tree Analysis 

**Figure 4:**
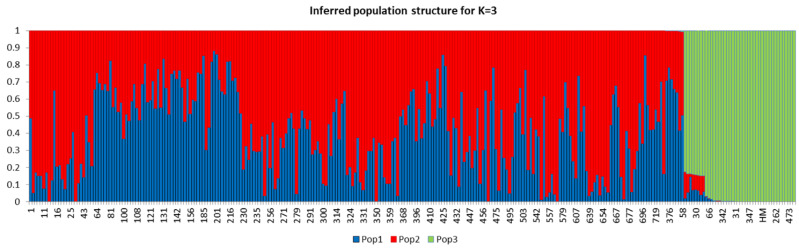
Population structure of 288 eggplant germplasms based on SNPs markers (5000, randomly selected). At K = 3, the population were estimated to be three (Pop1, Pop2, Pop3) based on STRUCTURE analysis.

**Figure 5:**
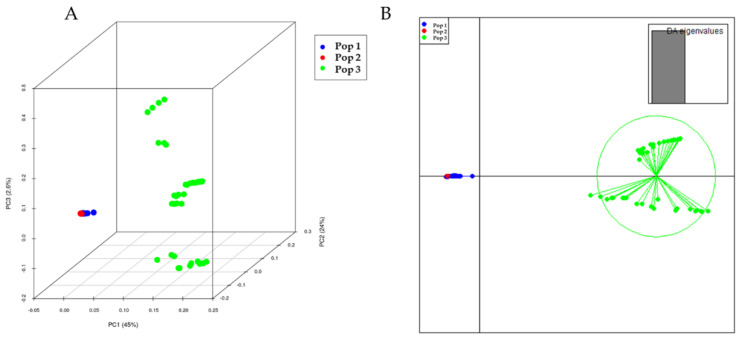
The 3D PCA (**A**) and DAPC (**B**) of 288 eggplant genetic resources based on 114,981 SNPs. Each color represents the population inferred by the structure program. The first three principal components; PC1, PC2, and PC3 accounted for 45%, 24% and 26.6%, respectively.

**Figure 6:**
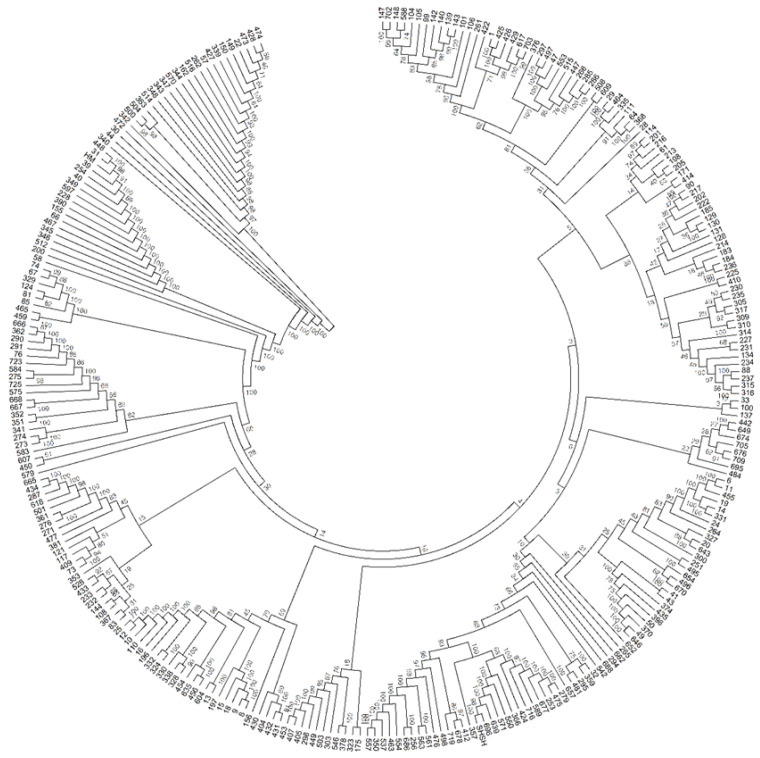
The evolutionary history was inferred using the Neighbor-Joining method. The optimal tree with the sum of branch length = 5.02 is shown. The evolutionary distances were computed using the Maximum Composite Likelihood method and are in the units of the number of base substitutions per site. Bootstrap percentages test of 1000 replicates are shown next to the branches. The analysis involved 288 nucleotide sequences. All ambiguous positions were removed for each sequence pair. There was a total of 114,981 positions in the final dataset. Evolutionary analyses were conducted in MEGA6. Each number/code outside the branch represents the eggplant accessions.

The population structure of the 288 eggplant genetic resources was inferred using STRUCTURE (v. 2.3.4) software (Pritchard et al., 2000). Admixture model-based simulations were carried out by varying K from 1 to 10 with 10 iterations. The estimated likelihood (lnP (D)) was greatest for K = 3 (Supplementary Figure S1), suggesting the presence of three main populations in the eggplant genetic resources panel (Figure 4). The PCA and DAPC of the eggplant population were analyzed and presented in Figure 5A,B. The PCA showed that the first three components comprised approximately 71.6% of the total variation and allowed the population to be categorized into three groups. The first PC comprised 45%, whereas the second and third comprised 24% and 2.6%, respectively. The eggplant genetic resources population was divided into three groups (blue, red, and green) as presented in the PCA and DAPC. Supplementary Table S2 contains information on the Admixture groups. The neighbor-joining (NJ) analysis of the entire population (288 eggplant accessions) is presented in Figure 6. As shown in the phylogenetic tree, many clusters were formed based on 114,981 SNPs.

### 2.4. Genome-Wide Association Analysis 

**Figure 7:**
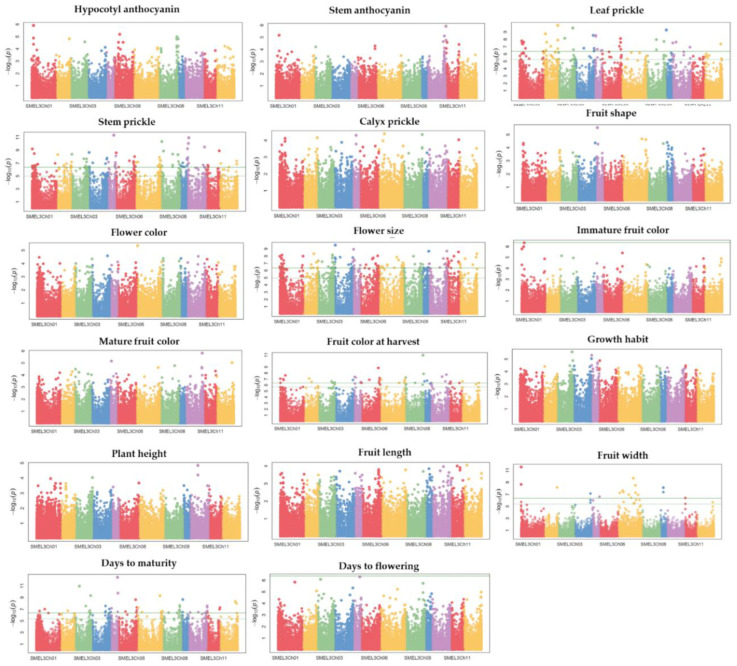
Manhattan plot depicting the association of 17 agro-morphological traits using 288 eggplant genetic resources. Each dot represents a single SNP, with the X-axis showing genomic location (chromosomes: colored and labeled) and Y-axis showing association level. The horizontal, green line represents the cut-off of the significant association.

A genetic association study was conducted to identify SNPs associated with qualitative and quantitative agro-morphological traits. The GWAS results of 17 agro-morphological traits were visualized in Manhattan (Figure 7) and QQ plots (Supplementary Figure S2). Among the 17 agro-morphological traits, significantly associated SNPs were found for six traits (Supplementary Table S3 and Figure 7). The Bonferroni-corrected threshold (-log *p* > 6.34) was used as a cut-off to identify marker-trait associations. A total of 377 significant SNPs associated with six agro-morphological traits were identified. These six traits (number of SNPs) were: days to maturity (51), flower size (121), fruit width (20), harvest fruit color (42), leaf prickles (38), and stem prickles (105). All SNPs significantly linked to six agro-morphological traits are presented in Supplementary Table S3. Among the significantly associated SNPs, the top 10 SNPs based on the log10 *p*-value for six agro-morphological traits are presented in Table 6. The largest fraction of significant SNPs (11.94%) was found on Ch01, followed by Ch07 and Ch06 with 11.67% and 10.08%, respectively. The smallest fraction of significant SNP markers (4.24% with 16 SNPs) was found on Ch12 for days to maturity (two), flower size (seven), fruit color at harvest (two), leaf prickle (one), and stem prickle (four). Except for Ch07 and Ch11, SNPs that were significantly associated with leaf prickles were found on all chromosomes. 

The number of significant SNPs associated with leaf prickles were seven on Ch02, six on Ch01 and Ch05, four on Ch04 and Ch06, three on Ch03, Ch08, and Ch10, and one on Ch09 and Ch11. Following flower size, the second highest number of significantly associated SNPs were found for stem prickles and located across all 12 chromosomes. The numbers of significantly associated SNPs with stem prickle found on Ch01, Ch08, Ch07, Ch10, and Ch05 were 14, 12, 11, 11, and 10, respectively. A relatively high number of significantly associated SNPs (121) were found for flower size across all 12 chromosomes. Of these SNPs, 15 were on Ch01, 14 on Ch04, 13 on Ch06 and Ch07, 12 on Ch03, and 11 on Ch10 and Ch11. Regarding fruit width, significantly associated SNPs were found only on a few chromosomes: Ch01 (three), Ch02 (one), Ch04 (one), Ch05 (two), Ch07 (nine), Ch09 (three), and Ch11 (one). Among the nine SNPs associated with fruit width located on Ch07, two of them were located in the intergenic region, and the other two were on genes that encode proteins with unknown functions. Among the SNPs associated with harvest color, seven were on Ch06, five on Ch01 and Ch07, and four on Ch03, Ch05, Ch08, and Ch10. Also, two SNPs were located on chromosomes Ch02, Ch09, Ch11, and Ch12. One SNP associated with harvest color was found in a gene that encodes sbt3, a subtilisin-like protease SBT3. Significantly associated SNPs with days to maturity were found on all chromosomes. Eight SNPs were located on Ch08, seven on Ch10, and six on Ch03 and Ch07 each. Relatively few SNPs associated with days to maturity were found on Ch05, Ch11, Ch01, Ch09, Ch12, and Ch02.

## 3. Discussion

The genetic diversity of plant genetic resources (PGRs), which provide useful alleles linked to plant development and improvement, is critical for the conservation and utilization of germplasm conserved in a gene bank [21,22]. DNA molecular markers provide valuable information for analyzing genetic diversity, genetic relationships, population structure, and core collections in a variety of crop species [23,24,25,26,27,28]. Representative core collections have been selected in various crops using different sampling strategies and clustering methods [29,30,31,32,33,34]. The M strategy was reported to be a useful approach for selecting a core collection with high genetic diversity and a reasonable size [32]. In this study, a representative core collection was established by selecting 288 eggplant resources from 587 *Solanum* accessions for efficient germplasm management and further studies. The greater the genetic diversity of germplasm, the greater the likelihood of success in breeding desirable traits. Studying and understanding the association of agro-morphological trait variations with genetic variable sites may assist in the selection and transformation of desirable traits to develop new cultivars through breeding programs. Diverse agro-morphological variations (fruit and leaf) of eggplant germplasm were found in previous studies [35,36,37]. Similarly, in this study, eggplant genetic resources collected from different countries possessed diverse agro-morphological characteristics. The correlation between agro-morphological traits was estimated and a strong positive correlation was observed between some agro-morphological traits such as stem prickles and leaf prickles, days to flowering and days to maturity, and immature fruit color and mature fruit color.

SNP markers are regarded as potentially promising breeding tools for use in genetic mapping and marker-assisted selection since they can be scored in parallel experiments at a low cost [38]. SNP markers were utilized in this study to assess population structure metrics, phylogenetic trees, and marker-trait associations. The phylogenetic tree analysis was conducted, and the evolutionary relationships among germplasm were based on the SNPs presented in this study. Population structure and kinship analysis allowed the clustering of eggplant germplasm into three broad groups. The majority of the germplasms used in this study (240 germplasms) belonged to *S. melongena*. Population 1 (Pop1) and 2 (Pop2) were mainly germplasm belonging to *S. melongena*, and a few unknown (*S.* spp.) species were also clustered. As presented in the PCA and DAPC, the first two clusters did not separate from each other entirely. A few germplasms from one to five genotypes belonging to other species were clustered in Group 3 (42 germplasms). The possibility of genetic material hybridization (naturally or via breeders) and migration of genetic resources from place to place could be the reason for creating subpopulations within the same species.

Genome-wide association studies have proved its efficiency in finding genomic regions linked with economically important agronomical features in several crops, including wheat [39,40,41,42], eggplant [36], potato [43], and soybean [44,45]. There are important agro-morphological traits to be improved in eggplant, including the development of prickleless varieties. Although prickly varieties are preferred in some areas due to their perceived improved organoleptic quality, prickles are generally regarded as undesirable since they can puncture the skin of the fruits and are problematic during harvesting and storage [46]. Previous research on raspberry and blackberry prickles has revealed that they are epidermal tissue outgrowths of modified glandular trichomes (GTs); once the outermost cells become lignified, lignification continues inward and downward until the prickles become completely lignified and thus mature [47,48]. A phenotypic assessment of prickles in *Solanum viarum* Dunal indicated that they may be initiated by GTs or triggered by GT-derived signals [49]. Transcriptome studies in raspberry and *S. viarum* revealed several transcription factors (TFs) that may be involved in prickle development [49,50]. In this study, three SNPs in three transcription factor genes (Trihelix transcription factor ASIL2, Probable WRKY transcription factor 35, and Probable transcription factor At5g28040) were found to have a significant association with stem prickles. One of the three SNPs was linked to both leaf and stem prickles. This SNP was located on Ch01 (14404622 bp) in a transcription factor gene (Trihelix transcription factor ASIL2). The SNP that was located on Ch05 (2527410 bp) was majorly found in eggplant genetic resources that have prickles on the stem. Several QTLs for prickle have been found in eggplant on chromosomes 2, 6, 7, and 8 [3,8,51,52]. A recent work genetically located a *Pl* locus on chromosome 6, and produced a 0.5 kb presence/absence variant marker for prickleless eggplant selection [53]. 

Interestingly, one SNP on Ch01 was found to be strongly linked with fruit color at harvest and was situated in a gene that produces the acetylserotonin O-methyl transferase (ASMT) enzyme. ASMT was also involved in a variety of plant growth and development dynamics. ASMT is the final enzyme in melatonin biosynthesis and may have a rate-limiting role in plant melatonin production. Several studies in recent years have confirmed that tryptophan decarboxylase (TrpDC), tryptamine 5-hydroxylase (T5H), serotonin N-acetyltransferase (SNAT), and acetylserotonin-O-methyltransferase (ASMT) are involved in melatonin synthesis in plants [54,55]. Sun et al. found that an exogenous melatonin treatment promoted ripening and improved tomato fruit quality after harvest [56]. Similarly, exogenous melatonin induced strawberry ASMT expression and accelerated strawberry fruit ripening via the ABA pathway [57]. Melatonin-deficient ASMT rice, on the other hand, showed accelerated senescence in detached flag leaves as well as a significantly lower yield [58].

In a previous study, it was indicated that the width and length of each flower organ affect the entire flower size [59]. Also, another study showed flower disc diameter was positively correlated with disc area in sunflower [60]. Among the total of 121 SNPs associated with flower size, 22 SNPs were found in the intergenic regions and others were in protein-coding genes with known (82 SNPs) and unknown (17 SNPs) functions. In this study, 20 SNPs significantly associated with fruit width were found. In a previous study, seven SNPs were identified on Ch01 (1), Ch02 (2), Ch03 (1), Ch09 (1), and Ch12 (1) that were linked with tomato fruit width (two) [61]. Some of the most significantly associated SNPs with flower size were found in genes encoding pentatricopeptide repeat-containing protein At5g14770, probable histone chaperone ASF1A, Ultraviolet-B receptor UVR8, MACPF domain-containing protein At1g14780, G2/mitotic-specific cyclin-1, two-component response regulator ORR21, and adenosine triphosphatase (ARSA1 ATPase) (Table 6).

The number of days needed until maturity is an important agronomic trait to determine and select early and late mature crops. The early flowering plant had a shortened maturity period as supported by a strong positive correlation of days to flowering and days to maturity (r = 0.64***). In previous studies, several SNP markers associated with days to maturity have been found in different crops, such as Kersting’s groundnut [62]. In this study, a total of 51 SNPs were associated with days to maturity, and one SNP was located in a gene that codes for pentatricopeptide repeat-containing protein (PPR). Mutations in these PPR protein-coding genes lead to the dysfunction of mitochondria and/or chloroplasts, thereby resulting in growth retardation, pollen abortion, and seed development defects in plants [63], indicating the important roles of PPR proteins in plant growth and development [64]. As presented in Table 6, some of the highly significantly associated SNPs with days to maturity were found in genes that encode DNA ligase 4 (LIG4) (Ch03 at 2.5 Mbp), PPL1 PsbP-like protein 1 chloroplastic (Ch03 at 8.8 Mbp), 4-coumarate--CoA ligase-like 5 (4CLL5) (Ch05 at 3.8 Mbp), Actin-7 (Ch05 at 4.0 Mbp), PHYC Phytochrome C (Ch07 at 126.0 Mbp), and PAL5 phenylalanine ammonia-lyase. DNA ligase enzymes perform crucial roles in DNA replication and repair processes by catalyzing the joining of adjacent polynucleotides [65]. Eukaryotes have multiple DNA ligases with unique roles in DNA metabolism, with clear differences in the functions of DNA ligase orthologues in mammals, yeast, and plants. DNA ligase 4 (LIG4) is found in all eukaryotes and facilitates the final step in the DSB repair pathway known as non-homologous end joining (NHEJ) [65]. Waterworth et al. [66] studied the role of DNA ligases in seed germination in terms of vigor and viability after storage under suboptimal conditions, as seen in much of the developing world. The identification of DNA repair mechanisms critical for rapid germination and seed lifespan can help forecast seed lot storage and germination performance, and these DNA repair pathways represent prospects for crop development with improved seed storability and germination performance features [66]. The other three SNPs were also found to be significantly associated with days to maturity and are located on Ch06 (9.7 Mbp) and Ch12 (2.6 Mbp and 9.3 Mbp) in genes that encode proteins with unknown functions (Table 6).

## 4. Materials and Methods

### 4.1. Plant Materials and Establishment of Eggplant Core Collection

A total of 587 eggplant resources collected from 50 countries, including 80 resources in the Philippines, 44 resources in China, and 16 resources in Korea, were used to establish a core collection. These germplasms belong to different species. The eggplant seedlings (eight to ten in triplicates) were planted in the National Agrobiodiversity Center (NAC) greenhouse at the Rural Development Administration (RDA), Jeonju, the Republic of Korea in 2021. The eggplants were cultivated according to the RDA-recommended eggplant cultivation method.

To establish a core collection, 52 microsatellite markers (single sequence repeats: SSRs) were used along with 17 morphological traits. Among 587 eggplant resources, a representative 288 resources were selected as a core collection based on the advanced maximization (M) strategy using a modified heuristic algorithm implemented in PowerCore software [67]. This core collection was further used in this study to evaluate genetic-phenotypic associations. Supplementary Table S4 contains information on the 52 SSR primers. Supplementary Table S5 shows the introduction number (IT), species name, and geographic origin of the 288 eggplant core collection used in this study.

### 4.2. Phenotyping

A total of 17 agro-morphological traits were assessed. This includes hypocotyl anthocyanin, stem anthocyanin staining, growth habit, plant height (cm), stem-prickle, leaf prickle, calyx prickle, flower size, flower color, fruit length (cm), fruit width (cm), fruit shape, immature fruit color, mature fruit color, harvest fruit color, days to flowering, and days to maturity. Except for the quantitative parameters, the scales or scores were used to assess the agro-morphological differences of eggplant genetic resources. The agro-morphological characterization descriptions are presented in Supplementary Table S6.

### 4.3. DNA Extraction and Genotyping-by-Sequencing (GBS)

DNA was extracted from the samples using a Genomic DNA Prep Kit (Inclone Biotech, Korea) following the manufacturer’s instructions. The GBS libraries were created using the restriction enzyme ApeKI (5′-GCWGC-3′) and a protocol modified from previous research [68]. Oligonucleotides containing the top and bottom strands of each barcode adapter and a common adapter were diluted (separately) with TE (50 μM each) and annealed with a thermocycler. DNA samples (100 ng/L) were added to individual adapter-containing wells. Samples (DNA with adapters) were digested overnight at 75 °C with ApeKI (New England Biolabs, Ipswich, MA, USA). The digested DNA samples, each with a specific barcode adapter, were pooled (5 μL each) and purified using a commercial kit (QIAquick PCR Purification Kit; Qiagen, Valencia, CA, USA) according to the manufacturer’s protocol. Restriction fragments from each library were then amplified in 50 μL volumes containing 2 μL of pooled DNA fragments, HerculaseII Fusion DNA Polymerase (Agilent, CA, USA), and 25 pmol each of the following primers: (A) 5′-AATGATACGGCGACCACCGAGATCTACACTCTTTCCCTACACGACGCTCTTCCGATCT-3′ and (B) 5′-CAAGCAGAAGACGGCATACGAGATCGGTCTCGGCATTCCTGCTGAACCGCTCTTCCGATCT-3′.

Barcode sequences were used to perform demultiplexing. Adapter trimming was done using cutadapt (version 1.8.3) [69], and sequence quality was trimmed using DynamicTrim and LengthSort of the SolexaQA program (v.1.13) [70]. DynamicTrim cuts low-quality bases at both ends of short reads according to the Phred score and refines it with high-quality cleaned reads. LengthSort removes excess base cuts made in DynamicTrim; Phred score of Dynamic-Trim ≥ 20, and LengthSort using short read lengths ≥ 25 bp. BWA (Burrows-Wheeler Aligner, ver.0.6.1-r104) [71] generated cleaned reads passing the preprocessing process and performed mapping to the reference genome of *Solanum melongena* L. (https://solgenomics.net/ accessed on 19 September 2022). Mapping was a preliminary step to detect raw SNPs (In/Del) between the *S. melongena* genome (Eggplant genome consortium V4.1) and sequenced samples. 

### 4.4. SNP Calling and Filtering

Clean reads were mapped to the reference genome sequence, and the obtained SAM files were used to discover raw SNPs using SAMtools (0.1.16) [72] and extract consensus sequences. SNP validation was conducted using SEEDERS in-house script [73] before SNP detection; raw SNP detection was performed, and default values were used except for the following options: a minimum mapping quality for SNPs (−Q) of 30, minimum mapping quality for gaps (−q) of 15, minimum read depth (−d) of 3, minimum InDel score for nearby SNP filtering (−G) of 30, SNPs within INT bp around a gap to be filtered (−w) of 15, window size for filtering dense SNPs (−W) of 30, and maximum read depth (−D) of 165.

An integrated SNP matrix was obtained between samples to assess SNPs between the assessed objects. A list of unions was generated by comparing each sample’s raw SNP sites to a standard template, and a non-SNP locus was filled in from the sample’s consensus sequence. The final SNP matrix was formed by filtering out the miscalled SNP sites using SNP comparison among samples. Based on the position, SNPs were classified as homozygous (SNP read depth ≥ 90%), heterozygous (40% ≤ SNP read depth ≤ 60%), etc., (homozygous/heterozygous; could not be separated by type). Based on the location information of the reference genome sequence (*Solanum melongena* L), the designated SNP positions were defined as “intergenic or genic regions”, and the genic region was further classed as “CDS or intron regions”. 

### 4.5. Population Structure and Phylogenetic Tree Analysis 

Population structure analysis was conducted using STRUCTURE software [74,75]. From 114,981 filtered SNPs loci of 288 genetic resources, 5,000 SNP loci at a level usable for analysis were randomly selected (six selected groups). Bayesian model-based approach with 10,000 burns in the period and 10,000 Markov Chain Monte Carlo (MCMC) was proposed. To find an appropriate K (population), K values were set from 1 to 10, and the number of iterations was 10. A web-based STRUCTURE HARVESTER [76] was used to determine the number of populations in the eggplant genetic resources panel. The appropriate K value was determined through the Delta K (ΔK) method [77]. The principal component analysis (PCA) [78] and discriminant analysis of principal components (DAPC) [79] were analyzed using the R program (SNPRelate and adegenet package).

The neighbor-joining method was used to infer the evolutionary history of the eggplant genetic resources using SNPs. The percentage of replicate trees in which the same clusters were formed, as determined by bootstrapping analysis (1000 replicates), is shown next to the branches. The tree is drawn to scale, with branch lengths in the same units as the evolutionary distances used to infer the phylogenetic tree. The evolutionary distances were calculated using the maximum composite likelihood method and are expressed in terms of the number of base substitutions per site. The analysis included 288 eggplant nucleotide sequences with the final dataset containing 114,981 SNPs. MEGA6 [80] was used to perform evolutionary analyses using the neighbor-joining method.

### 4.6. Genome-Wide Association Analysis and Candidate Genes Mining 

Association analysis was performed using 114,981 union SNP datasets using a mixed linear model (MLM) [81] implemented with an R package called the genomic association and prediction integrated tool (GAPIT) [82]. The significant threshold after Bonferroni correction was 4.35 × 10^−7^ (0.05/114981). The candidate genes were identified using the BLAST searching tools for each SNP in the eggplant genome database. 

### 4.7. Statistical Analysis 

Data summarization and descriptive statistics on agro-morphological data were performed using the Microsoft Excel program. PCA, DAPC, and correlation were all performed using the R program (version 4.2.1). Other software programs used in this study are discussed in the Section 4.

## 5. Conclusions

Crop breeding schemes require important agro-morphological characteristics. Phenotypic characterization is a time-consuming process and may not be precise enough for selection when a huge germplasm pool is available for breeding. The study of genetic association with morphological variation and the identification of SNPs associated with key agro-morphological and yield-related traits are important for assisting the selection process with rapid and accurate prediction. This study provides a comprehensive result on the association of genetic and agro-morphological traits of eggplants and significantly associated SNP markers for six eggplant agro-morphological traits are presented. A total of 377 significantly associated SNPs were found for stem prickles, leaf prickles, flower size, fruit width, fruit color at harvest, and days to maturity. These SNPs can be used for further research and to identify markers with high efficiency. We recommend further exploring the genes’ functions where SNPs are found for a better understanding of the molecular mechanisms of agro-morphological variation in eggplant germplasm.

## Figures and Tables

**Table 1:** The summary of qualitative agro-morphological traits of 288 eggplant collection.

Traits	Frequency	Percentage	Traits	Frequency	Percentage
**Hypocotyl anthocyanin**			**Growth habit**		
Absent	58	20.14	Upright	15	5.21
Present	230	79.86	Intermediate	260	90.28
**Stem anthocyanin**			Prostrate	13	4.51
Absent	202	70.14	**Flower size**		
Present	86	29.86	Small (<2 cm)	33	11.46
**Stem prickle**			Medium (2–3 cm)	250	86.81
Absent	266	92.36	Large (> 3 cm)	5	1.74
Present	22	7.64			
**Leaf prickle**			**Flower color**		
Absent	257	89.24	White	47	16.32
Present	31	10.76	Purple	176	61.11
**Calyx prickle**			Light purple	61	21.18
Absent	168	58.33	White and purple (mixed)	4	1.39
Present	120	41.67			
**Fruit shape**			**Mature fruit color**		
Round	80	27.78	Green	65	22.57
Oval	29	10.07	White	24	8.33
Ovate	15	5.21	Green-purple	35	12.15
Pear type	10	3.47	Light purple (more white)	16	5.56
Club	94	32.64	Purple	116	40.28
Elliptical	49	17.01	Orange	15	5.21
Cylindrical	11	3.82	Yellow	17	5.90
**Immature fruit color**			**Harvest color (ripening)**		
Green	110	38.19	Tan (pale brown)	136	47.22
White	10	3.47	Yellow	108	37.50
White-purple (advanced purple)	20	6.94	Green	3	1.04
Green-purple	41	14.24	Green purple	6	2.08
Purple	104	36.11	Light purple	2	0.69
Yellow	2	0.69	Purple	9	3.13
White-purple (advanced white)	1	0.35	Red	24	8.33

**Table 2:** The summary of quantitative agro-morphological traits of 288 eggplant collection.

Traits	No. of Germplasm	Min	Max	Average	SD
Plant height (cm)	288	13.20	209.10	87.76	24.20
Fruit width (cm)	288	0.10	25.05	5.77	2.28
Fruit length (cm)	288	0.60	37.22	16.80	7.54
Days to flowering	288	27	110	60.03	12.47
Days to maturity	288	88	156	111.57	9.24

**Table 3:** Summary of sequencing raw data.

Set	Sequencing File	No. of Barcode	No. of Sample	No. of Reads	Avg. Length (bp)	Total Length (bp)	GC(%)*1	Q30(%)*2	No. of Demultiplexed Reads (%)
Set1	R1	96	96	372,814,189	151	56,294,942,539	45.37	92.99	692,370,340 (92.86%)
	R2			372,814,189	151	56,294,942,539			
Set2	R1	96	96	371,354,724	151	56,074,563,324	45.91	92.43	722,810,406 (97.32%)
	R2			371,354,724	151	56,074,563,324			
Set3	R1	96	96	371,510,483	151	56,098,082,933	45.22	92.5	700,525,094 (94.28%)
	R2			371,510,483	151	56,098,082,933			
	Total	288	288	2,231,358,792		336,935,177,592			

**Table 4:** Raw and trimmed sequence statistics.

	Average/Plant	Total
Sum of raw reads	7,346,200.83	2,115,705,840
Total length of raw reads	1,109,276,325.83	3.19472 × 10^11^
Sum of trimmed reads	6,710,718.03	1,932,686,794
Total length of trimmed reads (bp)	761,869,536.23	2.19418 × 10^11^
Avg. length of trimmed reads (bp)	113.32	
Trimmed/Raw (%)	91.34%	
Sum of trimmed reads	6,710,718.03	1,932,686,794
No. of mapped reads	6,358,092.97	1,831,130,776
Percent of mapped reads (%)	94.62%	
No. of mapped region	84,273.83	24,270,863
Avg. depth of mapped region (#)	25.41	
Median depth of mapped region (#)	9.57	
Total length of mapped region (bp)	19,439,752.74	5,598,648,789
Avg. length of mapped region (bp)	228.16	
Reference Genome coverage (%)	1.7011%	

**Table 5:** Statistics of SNP filtering process.

Filter Stage	Filter Item	No. of SNPs
1	Total SNP	1,859,683
2	MAF (minor allele frequency) >5% *^1^	618,245
3	Missing data <30% *^2^	692,147
4	Missing data <30% & MAF >5%	114,981

(*1) MAF (minor allele frequency) >5%: SNPs with a minor allele frequency greater than 5% are selected from all samples of the locus. (*2) Missing data <30%: SNPs with missing data less than 30% were selected from all samples of the left.

**Table 6:** Top 10 significantly associated SNPs with leaf prickles, stem prickles, flower size, fruit width, fruit color at harvest and days to maturity.

	Chromosomes	Position (bp)	Ref.	Alt.	-Log (*p*-Value)	Genic/ Intergenic	Gene ID	Feature	Description
Leaf prickle	Ch02	79092061	T	C	9.9	Intergenic	-	-	-
	Ch02	10028712	A	C	8.75	Intergenic	-	-	-
	Ch03	80612399	T	A	9.54	Genic	SMEL_003g184780.1.01	exon,CDS	Protein of unknown function
	Ch03	35561623	C	T	8.14	Genic	SMEL_003g176660.1.01	Intron	ARF ADP-ribosylation factor 2
	Ch04	98734254	T	C	8.54	Genic	SMEL_004g217730.1.01	Intron	Protein of unknown function
	Ch05	7207267	C	T	8.52	Genic	SMEL_005g229060.1.01	exon,CDS	DPMS1 Dolichol-phosphate mannosyltransferase subunit 1
	Ch05	7552127	G	A	8.43	Intergenic	-	-	-
	Ch06	102955264	C	T	8.11	Genic	SMEL_006g264280.1.01	exon,CDS	Protein of unknown function
	Ch08	54103350	T	A	7.96	Genic	SMEL_008g305950.1.01	exon,CDS	MAG5 Protein transport protein SEC16A homolog
	Ch09	556607	C	T	9.27	Genic	SMEL_009g320070.1.01	Intron	At5g64680 Mediator-associated protein 2
Stem prickle	Ch01	1564720	T	G	9.19	Genic	SMEL_001g116710.1.01	Intron	PPRD2 Polyprenol reductase 2
	Ch04	433115	A	T	8.65	Intergenic	-	-	-
	Ch05	31730496	A	G	11.32	Genic	SMEL_005g234070.1.01	exon,CDS	GSTT1 Glutathione S-transferase T1
	Ch08	4114368	C	T	10.33	Genic	SMEL_008g300170.1.01	Intron	PCMP-H12 Pentatricopeptide repeat-containing protein At1g08070, chloroplastic
	Ch08	13353668	T	C	8.87	Genic	SMEL_008g302900.1.01	Intron	APY2 Apyrase 2 (Arabidopsis thaliana OX = 3702)
	Ch10	8483799	T	G	10.92	Genic	SMEL_010g341450.1.01	Intron	ETFA Electron transfer flavoprotein subunit alpha, mitochondrial
	Ch10	6032590	C	T	10.05	Genic	SMEL_010g340650.1.01	exon,CDS	MAA3 Probable helicase MAGATAMA 3
	Ch10	95813547	C	T	9.48	Genic	SMEL_010g353430.1.01	exon,CDS	ECI1 Enoyl-CoA delta isomerase 1, peroxisomal
	Ch10	914775	G	T	9.23	Genic	SMEL_010g336590.1.01	exon,CDS	BRO1 Vacuolar-sorting protein BRO1
	Ch11	71457233	A	T	8.9	Genic	SMEL_011g379480.1.01	Intron	RPL35 60S ribosomal protein L35
Flower size	Ch01	12681936	C	T	8.15	Genic	SMEL_001g126710.1.01	exon,CDS	Pentatricopeptide repeat-containing protein At5g14770, mitochondrial
	Ch02	59617555	G	A	8.31	Genic	SMEL_002g159930.1.01	exon,CDS	ASF1A Probable histone chaperone ASF1A
	Ch03	85061794	T	C	8.16	Intergenic	-		-
	Ch04	4151064	T	C	9.52	Genic	SMEL_004g202900.1.01	Intron	UVR8 Ultraviolet-B receptor UVR8
	Ch05	4580830	T	G	8.93	Genic	SMEL_005g227340.1.01	exon,CDS	UVR8 Ultraviolet-B receptor UVR8
	Ch09	34681620	A	T	8.67	Genic	SMEL_009g335180.1.01	exon,CDS	MACPF domain-containing protein At1g14780
	Ch09	34681628	A	C	8.67	Genic	SMEL_009g335180.1.01	exon,CDS	MACPF domain-containing protein At1g14780
	Ch10	103432323	A	C	8.69	Genic	SMEL_010g358670.1.01	Intron	G2/mitotic-specific cyclin-1
	Ch11	64268771	C	G	8.56	Genic	SMEL_011g374540.1.01	exon,CDS	RR21 Two-component response regulator ORR21
	Ch12	98996000	G	T	8.32	Genic	SMEL_012g398260.1.01	Intron	ARSA1 ATPase ARSA1
Fruit width	Ch01	759741	C	T	11.56	Genic	SMEL_001g115700.1.01	exon,CDS	UBC23 Probable ubiquitin-conjugating enzyme E2 23
	Ch01	771722	G	T	8.67	Genic	SMEL_001g115720.1.01	Intron	PIP2-7 Aquaporin PIP2-7
	Ch01	769956	G	C	8.64	Genic	SMEL_001g115710.1.01	exon,CDS	Protein of unknown function
	Ch02	76635827	T	A	8.16	Genic	SMEL_002g162940.1.01	exon,CDS	MTERF6 Transcription termination factor MTERF6, chloroplastic/mitochondrial
	Ch07	89696723	A	G	9.69	Genic	SMEL_007g282970.1.01	exon,CDS	Protein of unknown function
	Ch07	101888117	C	A	8.51	Genic	SMEL_007g283780.1.01	exon,CDS	Protein of unknown function
	Ch07	25532159	G	A	7.62	Genic	SMEL_007g277290.1.01	Intron	Similar to Sucrose synthase
	Ch07	31284604	A	T	7.38	Intergenic	-	-	-
	Ch09	15705642	C	T	8.13	Genic	SMEL_009g325830.1.01	exon,CDS	Protein of unknown function
	Ch09	15705607	G	A	8.1	Genic	SMEL_009g325830.1.01	exon,CDS	Protein of unknown function
Fruit color at Harvest	Ch01	30200788	T	A	7.61	Genic	SMEL_001g137250.1.01	exon,CDS	Protein of unknown function
	Ch05	4580830	T	G	7.35	Genic	SMEL_005g227340.1.01	exon,CDS	UVR8 Ultraviolet-B receptor UVR8
	Ch05	10921833	A	G	7.26	Intergenic	-	-	-
	Ch06	96640955	C	T	8.84	Genic	SMEL_006g258500.1.01	Intron	CLPX3 CLP protease regulatory subunit CLPX3, mitochondrial
	Ch06	103417425	G	A	7.17	Genic	SMEL_006g264870.1.01	exon,UTR	At1g51745 Uncharacterized protein At1g51745
	Ch08	101047764	C	T/C	10.96	Genic	SMEL_008g313940.1.01	exon,CDS	Protein of unknown function
	Ch08	104699628	G	G/A	7.87	Genic	SMEL_008g315650.1.01	Intron	YPTM2 GTP-binding protein YPTM2
	Ch08	2280526	C	T	7.27	Genic	SMEL_008g298990.1.01	exon,CDS	WRKY41 Probable WRKY transcription factor 41
	Ch10	89790523	A	A/G	7.74	Genic	SMEL_010g350710.1.01	CDS,exon	Protein of unknown function
	Ch10	64792041	G	G/T	7.41	Genic	SMEL_010g347400.1.01	Intron	Protein of unknown function
Days to Maturity	Ch01	48274449	A	G	7	Intergenic	-	-	-
	Ch03	25048685	G	A	10.94	Genic	SMEL_003g175720.1.01	Intron	LIG4 DNA ligase 4
	Ch03	88779347	A	G	9.34	Genic	SMEL_003g192220.1.01	Intron	PPL1 PsbP-like protein 1, chloroplastic
	Ch05	38653250	C	T	12.47	Genic	SMEL_005g238120.1.01	CDS,exon	4CLL5 4-coumarate--CoA ligase-like 5
	Ch05	40876927	C	G	9.75	Genic	SMEL_005g237880.1.01	exon,CDS	ACT7 Actin-7
	Ch06	97308270	C	T	8.62	Genic	SMEL_006g259010.1.01	exon,CDS	Protein of unknown function
	Ch07	126054497	G	A	9.31	Genic	SMEL_007g286710.1.01	exon,CDS	PHYC Phytochrome C
	Ch09	3651169	G	T	8.65	Genic	SMEL_009g321780.1.01	exon,CDS	PAL5 Phenylalanine ammonia-lyase
	Ch12	86643722	T	C	8.32	Genic	SMEL_012g392350.1.01	exon,CDS	Protein of unknown function
	Ch12	93119092	C	T	8	Genic	SMEL_012g394590.1.01	exon,CDS	Protein of unknown function

## Data Availability

The datasets generated and analyzed for the current study are available in the supplementary file, and the remaining datasets are accessible upon reasonable request from the corresponding author.

## Supplementary Materials

The following supporting information can be downloaded at: https://www.mdpi.com/2223-7747/11/19/2627/s1, Figure S1: Delta K values for K ranging from 1 to 10. The best fitting K values (K = 3) was selected; Figure S2: The QQ plots resulting from GWAS analysis for 17 agro-morphological traits 288 eggplant genet; Table S1: Summary of reference genome; Table S2: Population structure result (K = 3); Table S3: Significantly associated SNPs (377) for six agro-morphological traits; Table S4: 52 SSR primers used for selection of core collection; Table S5: The information of 288 eggplant germplasms; Table S6: Agro-morphological traits descriptions.
