# Supplementary material for: Genome-Wide Association Study for Agro-Morphological Traits in Eggplant Core Collection
Source: Plants (Basel). 2022 Oct 6;11(19):2627. doi: 10.3390/plants11192627 (PMC9571982; doi:10.3390/plants11192627)
Supplement: Supplementary file 1 [file plants-11-02627-s001.zip › Supplementary Figures.pdf]

Supplementary Figures

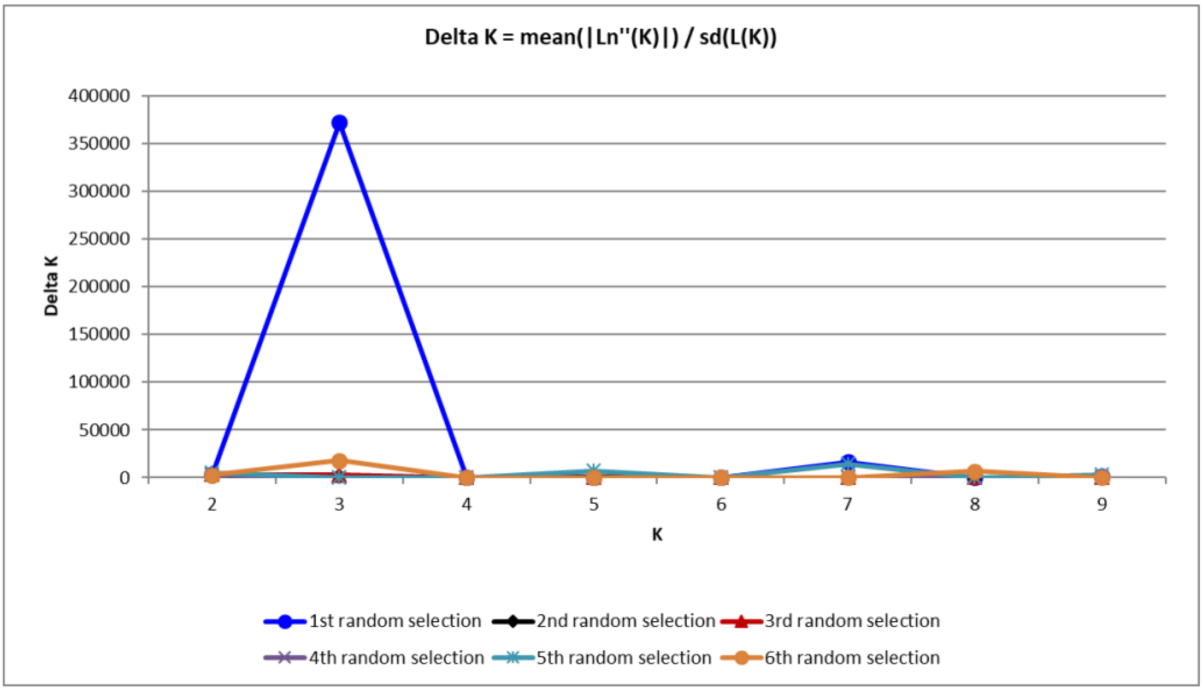

Figure S1. Delta K values for K ranging from 1 to 10 using the method proposed by Evanno et al. (2005). The best fitting K values (K=3) was selected.

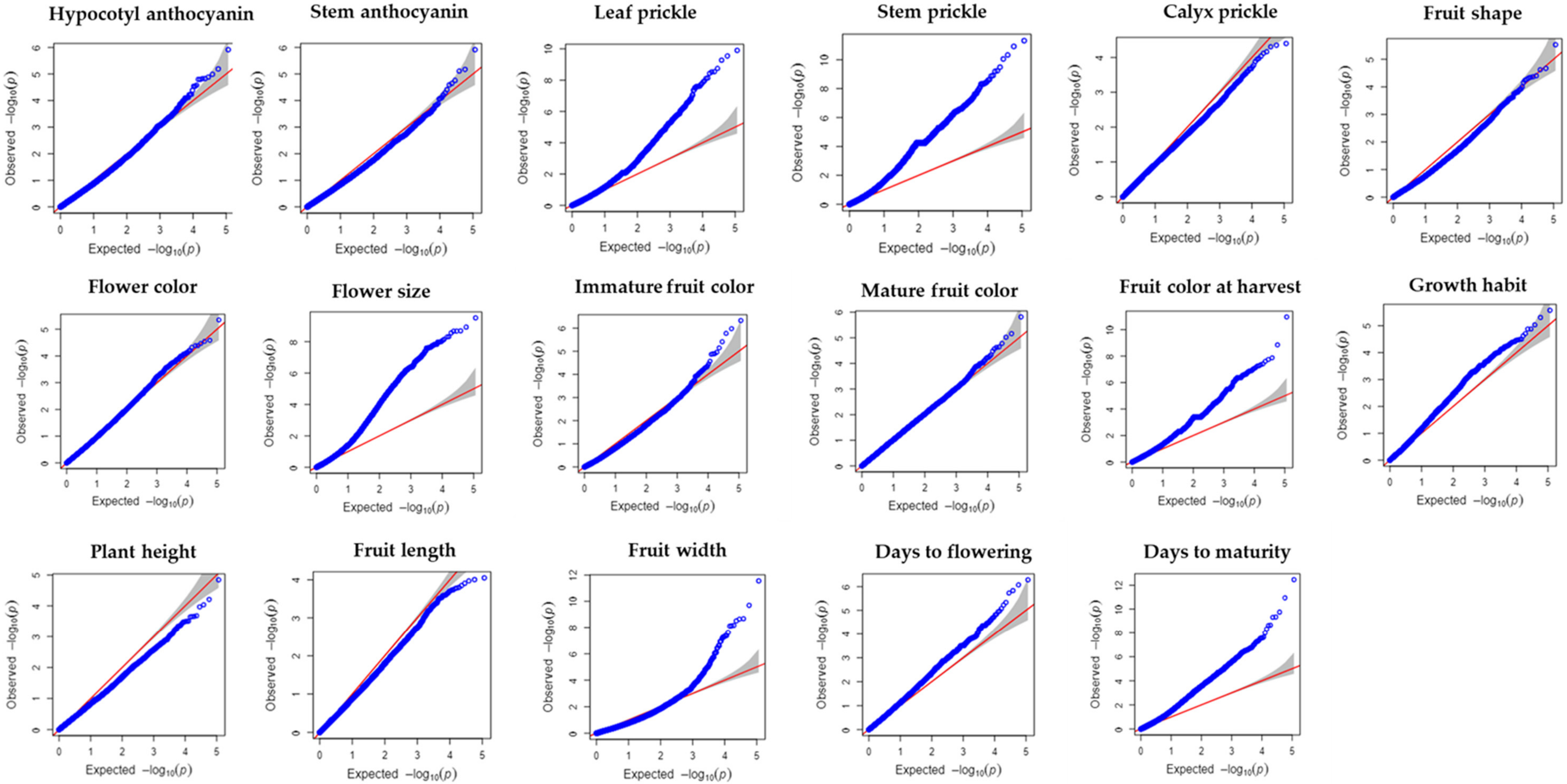

Figure S2. The QQ plots resulting from GWAS analysis for 17 morphological traits 288 eggplant genet.
