# Supplementary material for: Genome-Wide Association Study for Agro-Morphological Traits in Eggplant Core Collection
Source: Plants (Basel). 2022 Oct 6;11(19):2627. doi: 10.3390/plants11192627 (PMC9571982; doi:10.3390/plants11192627)
Supplement: Supplementary file 1 [file plants-11-02627-s001.zip › Supplementary Table S1.pdf]

Supplementary Table S1. Summary of reference genome.

Chromosome	Chromosome Length (bp)	No. of Transcript	Transcript Length (bp)	CDS length (bp)
SMEL3Ch01	136,534,347	3,838	5,405,349	4,566,804
SMEL3Ch02	83,340,400	1,569	1,977,381	1,682,649
SMEL3Ch03	97,014,425	3,092	4,350,282	3,648,759
SMEL3Ch04	105,671,276	2,373	3,158,701	2,670,972
SMEL3Ch05	43,853,053	1,861	2,589,410	2,186,841
SMEL3Ch06	108,971,373	2,878	3,750,129	3,139,461
SMEL3Ch07	142,382,611	2,634	3,282,716	2,823,408
SMEL3Ch08	109,575,448	2,223	2,807,949	2,402,763
SMEL3Ch09	36,100,499	1,642	2,299,425	1,943,238
SMEL3Ch10	106,644,908	2,591	3,344,260	2,812,460
SMEL3Ch11	72,290,491	1,823	2,426,398	2,068,614
SMEL3Ch12	100,421,161	1,911	2,414,803	2,033,247
**Total**	**1,142,799,992**	**28,435**	**37,806,803**	**31,979,216**
