# Supplementary material for: Genome-Wide Association Study for Agro-Morphological Traits in Eggplant Core Collection
Source: Plants (Basel). 2022 Oct 6;11(19):2627. doi: 10.3390/plants11192627 (PMC9571982; doi:10.3390/plants11192627)
Supplement: Supplementary file 1 [file plants-11-02627-s001.zip › Supplementary Table S5.pdf]

Table S5. The information of 288 eggplant germplasms.

Accession ID	Introduction (IT) Number	Species Name	Country of origin (Code)
1	102771	*Solanum melongena*	KOR
6	136552	*Solanum melongena*	NPL
8	136554	*Solanum melongena*	NPL
9	136555	*Solanum melongena*	NPL
10	136556	*Solanum melongena*	NPL
11	136557	*Solanum melongena*	NPL
12	136558	*Solanum melongena*	NPL
13	136559	*Solanum melongena*	NPL
14	136560	*Solanum melongena*	NPL
15	136561	*Solanum melongena*	NPL
16	136562	*Solanum melongena*	NPL
18	136564	*Solanum melongena*	NPL
19	136565	*Solanum melongena*	NPL
20	136566	*Solanum melongena*	NPL
22	136568	*Solanum melongena*	NPL
24	136570	*Solanum melongena*	IND
25	136571	*Solanum melongena*	IND
28	155182	*Solanum melongena*	KOR
29	160412	*Solanum melongena*	CHN
30	164930	*Solanum melongena*	UNK
31	164931	*Solanum macrocarpon*	UNK
32	164932	*Solanum melongena*	UNK
33	164933	*Solanum melongena*	UNK
39	170471	*Solanum melongena*	UNK
40	170472	*Solanum melongena*	UNK
41	170473	*Solanum melongena*	UNK
43	170475	*Solanum melongena*	UNK
44	170476	*Solanum* sp.	UNK
47	171343	*Solanum melongena*	CHN
49	180703	*Solanum melongena*	EGY
50	180718	*Solanum melongena*	EGY
57	189680	*Solanum melongena*	MYS
58	189681	*Solanum melongena*	UNK
61	189684	*Solanum melongena*	UNK
64	189687	*Solanum melongena*	UNK
66	189689	*Solanum melongena*	UNK
67	189690	*Solanum melongena*	UNK

73	189696	*Solanum melongena*	UNK
74	189697	*Solanum melongena*	UNK
76	189699	*Solanum melongena*	UNK
81	189704	*Solanum melongena*	UNK
83	189706	*Solanum melongena*	UNK
85	189708	*Solanum melongena*	UNK
88	189711	*Solanum melongena*	UNK
90	189713	*Solanum melongena*	UNK
99	189722	*Solanum melongena*	JPN
100	189723	*Solanum melongena*	JPN
101	189724	*Solanum melongena*	JPN
104	189727	*Solanum melongena*	JPN
105	189728	*Solanum melongena*	JPN
106	189729	*Solanum melongena*	JPN
108	189731	*Solanum melongena*	UNK
110	189733	*Solanum melongena*	UNK
111	189734	*Solanum melongena*	UNK
114	189737	*Solanum melongena*	UNK
117	189740	*Solanum melongena*	UNK
121	189744	*Solanum melongena*	UNK
124	189747	*Solanum melongena*	UNK
128	189751	*Solanum melongena*	UNK
129	189752	*Solanum melongena*	UNK
130	189753	*Solanum melongena*	UNK
131	189754	*Solanum melongena*	UNK
134	189757	*Solanum melongena*	UNK
137	189760	*Solanum melongena*	JPN
139	189762	*Solanum melongena*	JPN
140	189763	*Solanum melongena*	JPN
142	189765	*Solanum melongena*	JPN
143	189766	*Solanum melongena*	JPN
144	189767	*Solanum melongena*	UNK
147	189770	*Solanum melongena*	JPN
148	189771	*Solanum melongena*	JPN
149	191637	*Solanum virginianum*	NPL
150	196171	*Solanum* sp.	PER
155	200146	*Solanum* sp.	NPL
156	200244	*Solanum melongena*	NPL
162	200513	*Solanum sp.*	NPL
171	201059	*Solanum melongena*	PHL

175	201063	*Solanum melongena*	UNK
183	201071	*Solanum melongena*	PHL
184	201072	*Solanum melongena*	PHL
185	201073	*Solanum melongena*	PHL
196	201084	*Solanum melongena*	PHL
197	201085	*Solanum melongena*	PHL
198	201086	*Solanum melongena*	PHL
200	201088	*Solanum melongena*	PHL
201	201089	*Solanum melongena*	PHL
202	201090	*Solanum melongena*	PHL
205	201093	*Solanum melongena*	PHL
213	201101	*Solanum melongena*	PHL
214	201102	*Solanum melongena*	PHL
216	201104	*Solanum melongena*	PHL
217	201105	*Solanum melongena*	PHL
222	201110	*Solanum melongena*	PHL
225	201113	*Solanum melongena*	PHL
227	201115	*Solanum melongena*	PHL
228	201116	*Solanum melongena*	PHL
230	201118	*Solanum melongena*	PHL
231	201119	*Solanum melongena*	PHL
232	201120	*Solanum melongena*	PHL
233	201121	*Solanum melongena*	PHL
234	201122	*Solanum melongena*	PHL
235	201123	*Solanum melongena*	PHL
236	201124	*Solanum melongena*	PHL
237	201125	*Solanum melongena*	PHL
251	203189	*Solanum melongena*	RUS
253	203191	*Solanum melongena*	GEO
254	203192	*Solanum melongena*	AZE
256	203194	*Solanum melongena*	MDA
261	207254	*Solanum melongena*	RUS
262	208410	*Solanum virginianum*	NPL
264	208417	*Solanum melongena*	IND
265	208418	*Solanum melongena*	IND
266	210126	*Solanum* sp.	UNK
271	218695	*Solanum melongena*	THA
273	218697	*Solanum melongena*	THA
274	218699	*Solanum melongena*	THA
275	218700	*Solanum melongena*	THA

276	218701	*Solanum melongena*	THA
279	218718	*Solanum melongena*	UZB
285	218771	*Solanum melongena*	CHN
286	218772	*Solanum melongena*	CHN
287	218774	*Solanum melongena*	THA
290	218778	*Solanum melongena*	THA
291	218779	*Solanum melongena*	THA
294	218790	*Solanum melongena*	RUS
295	218791	*Solanum melongena*	RUS
297	218810	*Solanum melongena*	UNK
298	218841	*Solanum melongena*	IND
300	219030	*Solanum melongena*	ARM
303	219123	*Solanum melongena*	IND
305	219125	*Solanum melongena*	PHL
309	219129	*Solanum melongena*	PHL
310	219130	*Solanum melongena*	PHL
314	219134	*Solanum melongena*	PHL
315	219135	*Solanum melongena*	PHL
316	219136	*Solanum melongena*	PHL
317	219137	*Solanum melongena*	PHL
323	219143	*Solanum melongena*	PRI
324	219144	*Solanum melongena*	IND
327	219147	*Solanum melongena*	IND
328	219148	*Solanum melongena*	BGD
329	219149	*Solanum melongena*	IDN
330	219150	*Solanum melongena*	BGD
331	219837	*Solanum melongena*	IND
332	219865	*Solanum melongena*	IND
335	220040	*Solanum melongena*	CHN
338	220043	*Solanum melongena*	BGD
339	220478	*Solanum americanum*	BTN
340	220606	*Solanum macrocarpon*	MYS
341	220607	*Solanum virginianum*	THA
342	220608	*Solanum sisymbriifolium*	UNK
343	220609	*Solanum americanum*	PHL
344	220610	*Solanum incanum*	PHL
345	220611	*Solanum incanum*	THA
346	220612	*Solanum capense*	BWA
347	220613	*Solanum villosum*	BGD
348	220614	*Solanum scabrum*	CMR

349	220615	*Solanum aethiopicum*	ZMB
350	221496	*Solanum melongena*	TUR
351	224124	*Solanum melongena*	LAO
352	224125	*Solanum melongena*	LAO
353	224126	*Solanum melongena*	LAO
357	224185	*Solanum melongena*	UKR
359	224187	*Solanum melongena*	AUS
361	224249	*Solanum melongena*	THA
362	224250	*Solanum melongena*	THA
363	224255	*Solanum pseudocapsicum*	UNK
366	224319	*Solanum melongena*	UZB
367	224419	*Solanum melongena*	MYS
368	224421	*Solanum melongena*	MYS
370	224439	*Solanum melongena*	UNK
374	224542	*Solanum melongena*	USA
376	224553	*Solanum melongena*	CHN
378	224589	*Solanum melongena*	IND
381	224600	*Solanum melongena*	IDN
390	224790	*Solanum melongena*	CHN
396	225008	*Solanum melongena*	UNK
404	225095	*Solanum melongena*	MMR
405	225096	*Solanum melongena*	MMR
407	225098	*Solanum melongena*	MMR
409	230117	*Solanum melongena*	IDN
410	230118	*Solanum melongena*	PHL
412	230120	*Solanum melongena*	TUR
414	230122	*Solanum melongena*	PHL
422	235385	*Solanum* sp.	KOR
424	235437	*Solanum* sp.	UZB
425	235438	*Solanum* sp.	KOR
426	235439	*Solanum* sp.	KOR
427	235491	*Solanum* sp.	NPL
428	235531	*Solanum virginianum*	NPL
429	235555	*Solanum macrocarpon*	UNK
430	235795	*Solanum* sp.	MMR
431	246777	*Solanum* sp.	MMR
432	246778	*Solanum* sp.	MMR
433	246779	*Solanum* sp.	MMR
434	247868	*Solanum melongena*	THA
435	250101	*Solanum melongena*	AUS

442	250108	*Solanum melongena*	ARM
447	250113	*Solanum melongena*	UNK
448	250114	*Solanum melongena*	KOR
449	250115	*Solanum melongena*	MMR
450	250116	*Solanum melongena*	MMR
453	260886	*Solanum* sp.	MMR
454	260910	*Solanum melongena*	IND
455	260911	*Solanum melongena*	IND
456	260912	*Solanum melongena*	IND
459	260915	*Solanum melongena*	IND
463	267559	*Solanum melongena*	TUR
464	277860	*Solanum melongena*	CHN
465	283345	*Solanum melongena*	IDN
467	283605	*Solanum melongena*	MMR
472	288851	*Solanum ptycanthum*	UNK
473	288858	*Solanum virginianum*	CAN
474	288866	*Solanum virginianum*	NPL
475	296993	*Solanum melongena*	KGZ
476	297123	*Solanum melongena*	UNK
477	297130	*Solanum melongena*	MMR
481	319719	*Solanum melongena*	RUS
484	319722	*Solanum melongena*	JPN
495	903175	*Solanum melongena*	RUS
496	903177	*Solanum melongena*	FRA
497	331870	*Solanum* sp.	UNK
498	907640	*Solanum* sp.	UNK
500	910827	*Solanum pseudocapsicum*	UNK
501	K003875	*Solanum melongena*	THA
503	K016532	*Solanum melongena*	IND
504	K019176	*Solanum pseudocapsicum*	DEU
508	K051476	*Solanum melongena*	CHN
512	K125042	*Solanum rigescentoides*	UNK
514	331869	*Solanum* sp.	MYS
515	K125055	*Solanum melongena*	CHN
516	K125079	*Solanum mammosum*	TWN
528	K126957	*Solanum melongena*	MMR
537	K134179	*Solanum melongena*	TUR
542	K139708	*Solanum melongena*	BGR
546	K142181	*Solanum melongena*	TWN
550	K142917	*Solanum melongena*	KGZ

553	K144638	*Solanum melongena*	UKR
554	K145105	*Solanum melongena*	TUR
557	K145198	*Solanum melongena*	TUR
561	K146835	*Solanum melongena*	BGR
563	K146852	*Solanum melongena*	BGR
570	K150362	*Solanum mammosum*	THA
571	K151033	*Solanum melongena*	UKR
575	K152983	*Solanum melongena*	LAO
579	K152993	*Solanum melongena*	LAO
583	K153001	*Solanum melongena*	LAO
584	K153003	*Solanum melongena*	THA
588	K154735	*Solanum melongena*	JPN
589	K154984	*Solanum melongena*	UZB
597	K160305	*Solanum melongena*	CHN
604	K166157	*Solanum melongena*	NPL
607	K168113	*Solanum melongena*	MMR
609	K169957	*Solanum melongena*	CHN
617	K171621	*Solanum melongena*	KOR
618	K175906	*Solanum melongena*	THA
635	K206627	*Solanum melongena*	IND
639	K208073	*Solanum melongena*	UNK
643	K225383	*Solanum melongena*	PAK
646	K226440	*Solanum melongena*	CHN
649	K226812	*Solanum melongena*	DEU
652	K226815	*Solanum melongena*	NAM
654	K226817	*Solanum melongena*	ESP
657	K226820	*Solanum melongena*	ESP
662	K226825	*Solanum melongena*	ESP
665	K243725	*Solanum mammosum*	THA
666	K243728	*Solanum mammosum*	THA
667	K243729	*Solanum mammosum*	THA
668	K243730	*Solanum mammosum*	THA
670	K247200	*Solanum melongena*	USA
674	K247206	*Solanum melongena*	DEU
676	K247208	*Solanum melongena*	UNK
677	K247210	*Solanum melongena*	UNK
678	K247211	*Solanum melongena*	RUS
686	K247758	*Solanum melongena*	UZB
688	K247894	*Solanum melongena*	TUR
695	K253800	*Solanum melongena*	THA

696	K253801	*Solanum melongena*	THA
702	K255474	*Solanum aethiopicum*	KOR
703	K257622	*Solanum melongena*	GEO
705	K257631	*Solanum melongena*	GEO
709	K257723	*Solanum melongena*	GEO
716	K261669	*Solanum melongena*	SYR
719	K261672	*Solanum melongena*	UNK
723	K261676	*Solanum melongena*	USA
725	K266333	*Solanum melongena*	GEO
HM	HM	*Solanum melongena*	UNK
SHSH	SHSH	*Solanum melongena*	UNK

Note: UNK: Unknown
