# Supplementary material for: Genome-Wide Association Study for Agro-Morphological Traits in Eggplant Core Collection
Source: Plants (Basel). 2022 Oct 6;11(19):2627. doi: 10.3390/plants11192627 (PMC9571982; doi:10.3390/plants11192627)
Supplement: Supplementary file 1 [file plants-11-02627-s001.zip › Supplementary Table S6.pdf]

Supplementary Table S6: Morphological traits descriptions.

No.	Traits	Code and Description
1	Hypocotyl anthocyanin	1: Absent 9: Present
2	Growth habit	1: upright 3: semi-upright (intermediate) 5: prostrate
3	Plant Height (cm)	
4	Stem anthocyanin	1: Absent 9: Present
5	Stem prickles	1: Absent 9: Present
6	Leaf prickles	1: Absent 9: Present
7	Calyx prickles	1: Absent 9: Present
8	Flower Size (cm)	1: Small (< 2 cm) 3: Medium (2-3 cm) 5: Large (> 3 cm)
9	Flower color	1: White 2: purple, 3: soft purple 4: white purple
10	Fruit length (cm)	
11	Fruit width (cm)	
12	Fruit shape	1: Round 2: Ovoid , 3: Ovate , 4: Pear type, 5: Club, 6: Ellipsoid, 7: Cylindrical
13	Immature fruit color	1. Green, 2. White 3. White-purple (advanced purple), 4. Green-purple, 5. Purple, 6. Yellow, 7. White-purple (advanced white)
14	Mature fruit color	1: Green, 2: White, 3: Green purple 4: white purple 5: Purple 6: Orange, 7: Yellow
15	Fruit color at harvest	1. Tan (pale brown), 2. Yellow, 3. Green, 4. Green purple, 5. Light purple, 6. Purple, 7. Red
16	Days to flowering	Number of days
17	Days to maturity	Number of days
